# Evolution of Cross-Neutralizing Antibody Specificities to the CD4-BS and the Carbohydrate Cloak of the HIV Env in an HIV-1-Infected Subject

**DOI:** 10.1371/journal.pone.0049610

**Published:** 2012-11-13

**Authors:** Iliyana Mikell, Leonidas Stamatatos

**Affiliations:** 1 Seattle BioMed, Seattle, Washington, United States of America; 2 Department of Global Health, University of Washington, Seattle, Washington, United States of America; University of Amsterdam, The Netherlands

## Abstract

Broadly neutralizing antibodies are considered an important part of a successful HIV vaccine. A better understanding of the factors underlying their development during infection and of the epitopes they target is needed to elicit similar antibody responses by vaccination. We and others reported that, on average, it takes 2 to 3 years for cross-reactive neutralizing antibodies to become detectable in the sera of HIV-1-infected subjects and that they target a limited number of epitopes on the HIV Envelope. Here we investigated the emergence and evolution of the earliest cross-reactive neutralizing antibody specificities in one HIV-1-infected individual, AC053. We defined two distinct epitopes on Env that are targeted by the broadly neutralizing antibody responses developed by AC053. The first specificity became evident at 3 years post infection and targeted the CD4-binding site of Env. Antibodies responsible for that specificity neutralized most, but not all, viruses susceptible to neutralization by the plasma antibodies of AC053. The second specificity became apparent approximately a year later. It was due to PG9-like antibodies, which were able to neutralize those viruses not susceptible to the anti-CD4-BS antibodies in AC053. These findings improve **our** understanding of the co-development of broadly neutralizing antibodies that target more than one epitope during natural HIV-1-infection in selected HIV+ subjects. They support the hypothesis that developing broadly neutralizing antibody responses targeting distinct epitopes by immunization could be feasible.

## Introduction

A neutralizing antibody (NAb) response of sufficient duration and magnitude is considered an important part of a successful HIV vaccine [1]–[3]. Numerous studies have demonstrated sterilizing protection by NAbs against challenge with simian-human immunodeficiency virus (SHIV) in nonhuman primate models [4]–[7], and the selection pressure that NAbs exert on the virus during natural infection in humans [8]–[11]. These observations overwhelmingly suggest that the presence of similar types of NAbs elicited by a vaccine would be beneficial to the vaccinee.

The only target for neutralizing antibodies on HIV is the virally encoded envelope glycoprotein (Env) spike. The functional unit of Env, as expressed on the surface of infectious virions, is a trimer of non-covalently-associated extracellular subunit (gp120) and transmembrane subunit (gp41). Due to the tremendous genetic diversity of the HIV Env, the antibodies elicited by a successful vaccine will have to neutralize a wide range of circulating HIV-1 isolates [2]. Such antibodies are referred to as broadly neutralizing antibodies (bNAbs). Although eliciting such responses by vaccination has not yet been achieved, numerous studies have investigated the development and characteristics of broadly neutralizing antibodies produced during natural HIV-1 infection in humans. Such studies provided novel information on the epitopes targeted by these cross-clade neutralizing activities, and the factors associated with their development. Several studies of infected subjects in early and chronic HIV-1 infection have demonstrated that broadly neutralizing antibody responses develop in approximately 15% of infected individuals [1], [12]–[18], and become detectable within 2 to 3 years post infection [14], [16], [19]. In contrast, autologous neutralizing antibody responses develop weeks to months after infection in virtually all infected subjects, but although potent, are largely strain-specific and rapidly escaped by the virus [8], [20]–[23].

Systematic analyses of the epitope specificities of broadly neutralizing antibody responses in HIV+ sera have demonstrated that a limited number of specificities are responsible for the serum cross-neutralizing activity in any given individual [13], [15], [24]–[29]. Monoclonal antibodies (MAbs) with broad neutralizing activities have been isolated from chronically-infected HIV+ subjects and have been shown to target structurally-conserved epitopes of Env: the CD4 binding site (CD4-BS) [30]–[34], conserved elements of the V2 loop and associated carbohydrates [35], [36] and conserved elements of the V3 loop and associated carbohydrates [37], [38] on gp120. In addition, a few broadly neutralizing MAbs target the membrane proximal external region of the gp41 subunit [39], [40].

In a previous study we sought to determine the timing of the development of the broadly neutralizing antibody response to HIV-1 clade B in a cohort of anti-retroviral naïve subjects that have been monitored longitudinally from a few months to up to 7 years post infection [14]. Our findings indicated that broadly neutralizing antibody responses emerged gradually, and became detectable at approximately 2.5 years of infection. Subsequently, these responses increased both in potency and breadth. Others have also reported on a similar time-dependent development of cross-neutralizing antibody responses during HIV-1 infection [16], [19], [41]. Epitope mapping studies of the polyclonal IgG responses in plasmas from the cohort we examined indicated that the earliest cross-neutralizing antibody responses targeted either the CD4-BS on gp120 or epitopes not present on monomeric gp120 [14]. Since neutralizing activities against the gp41 subunit of Env were not detectable in the plasmas, we assumed that these later neutralizing activities targeted epitopes present on the oligomeric Env, but not present on monomeric gp120. We also reported that in certain plasmas a small number of epitope specificities contributed to the overall cross-neutralizing activity of a plasma sample. For example, anti-CD4-BS antibodies were responsible for neutralizing a certain number of viruses, and antibodies that could not be mapped to gp120 were responsible for neutralizing different viruses against which the plasma was tested. Two recent studies demonstrated that, indeed, the overall cross-neutralizing activity in a chronically HIV-infected subject could be recapitulated by two monoclonal antibodies isolated from that subject, that target distinct epitopes on Env [42], [43]. However, those studies did not examine whether both specificities emerged at the same time or not, and how they evolved over time. In the present study we aimed to better understand the relative emergence and evolution of dual epitope specificities in a well-characterized case control from the above-described cohort.

## Materials and Methods

### Human Plasma Samples

Samples from subject AC053 from the Ragon Institute of Massachusetts General Hospital (MGH) ‘acute/early’ HIV infection cohort (also referred to as ‘primary’ cohort) were used in this study. The subject was infected with clade B HIV-1, had no AIDS-defining illnesses, and was not on antiretroviral therapy at the time of sample collection. In the MGH Acute HIV infection Cohort ‘primary infection’ was defined by detectable HIV RNA in the presence of either (i) a negative p24 ELISA or (ii) a positive ELISA but evolving WB, or (iii) documented negative HIV ELISA within past 6 months. The date of infection for AC053 was known and 9 samples were collected longitudinally starting at a 0.82 years post infection to 6.85 years post infection, after which CD4 T cell count fell below 200 and the subject was placed on antiretroviral therapy. Samples were heat-inactivated at 56°C for 1 hour before use in neutralization assays. The neutralizing activity of AC053 plasma has been reported by our group previously [14].

### Plasma Antibody Adsorptions to Monomeric gp120

Recombinant monomeric SF162 gp120 WT or SF162K160N gp120 proteins were coupled to MyOne Dynabeads Tosylactivated (Invitrogen) as previously described [13], [24]. Briefly, 50 mg of magnetic beads were reacted with 1 mg protein ligand overnight at 37°C with rotation. After collecting the beads on a magnet, the supernatant was removed and the beads were incubated overnight at 37°C in PBS, 0.5%BSA, 0.05% Tween 20. The magnetic beads were washed twice with PBS, 0.1%BSA, 0.05% Tween 20, and stored at 4°C in the same buffer, with the addition of 0.02% Sodium Azide. Bead-coupled Env proteins were tested for antigenic integrity by flow cytometry using known MAbs b12, 447-52D, 2G12, PG9, and 4E10, followed by detection with goat-anti-human-IgG-FITC secondary antibody (data not shown). Mock adsorption/elution experiments using several anti-HIV Env MAbs at a concentration of 10 µg/ml in naïve plasma were performed as a positive control. 250 µl or 500 µl of plasma diluted 1∶5 in DMEM/10%FBS were incubated with 100 µl or 200 µl Env protein-coupled beads, respectively, at 37°C for 120 min with gentle rotation. The samples were placed on a magnet and the beads were isolated. Four rounds of bead adsorptions were performed per sample.

The anti-gp120 plasma antibodies bound to the bead-coupled Env proteins were eluted in a series of increasingly acidic solutions as previously described [24]. The beads from each serial adsorption were combined and incubated in 0.1 M Glycine-HCl, pH 2.7 for 30 seconds with vortexing. The beads were collected by brief centrifugation and held in place by a magnet. The supernatant was removed and adjusted to pH 7.5 with 1 M Tris (pH 9.0). The process was repeated with the beads in 0.1 M Glycine-HCl, pH 2.3, and then again in pH 1.7. The final supernatants were buffer exchanged in PBS and washed over a 30 kD Amicon Ultra centrifugation concentrator (Millipore). Concentration of immunoglobulin was determined by absorbance at 280 nm (NanoDrop Spectrophotometer ND-1000, Thermo). The anti-gp120-antibody depleted plasmas and the anti-gp120 antibodies eluted from gp120-coated beads were tested by ELISA for binding activity, and for neutralizing activity (data not shown).

### Neutralization Assays

Single round-competent viruses expressing Env’s from Clades A, B, and C primary isolates were used in this study. The clade B SF162, JRFL and YU2 isolates were isolated during chronic HIV-1 infection, and the remaining isolates were isolated during acute infection [44]–[46]. Single-round competent viruses were produced in 293 cells as previously described [47], with the modification that GeneJuice (EMD Millipore) was used as the transfection reagent. In the cases when kifunenisne- or swainsonine-treated pseudoviruses were used, the 293 producer cells were treated with 50 µM of the glycosidase inhibitor after transfection and the pseudovirus was collected after 72 hour incubation. All treated pseudoviruses were tested for neutralization resistance to PG9 or PG16.

The neutralizing activities of plasmas were determined using the Tzm-bl-based neutralization assay [48]. Briefly, plasma dilutions were pre-incubated with single-round competent virions (pseudovirus) for 90 minutes at 37°C. The plasma/pseudovirus mixture was added to TZM-bl cells (plated the day before at 3000 cells per well in a 96-well plate) for 72 hrs at 37°C. The supernatant was removed and 100 µl of Steady-Glo Luciferase Assay Substrate (Promega) was added to each well. Plates were incubated for 15 minutes at room temperature away from light, and 75 µl of the lysate was transferred to micro titer plates. The cell-associated luciferase activity for each well was determined on a Fluoroscan Luminometer (Thermo). Percent neutralization was calculated at each plasma dilution as the percent inhibition of viral entry by the plasma sample compared to the absence of plasma. For each plasma/virus combination tested, a neutralization curve (percent neutralization versus plasma dilution) was generated using GraphPad Prism 5 (GraphPad Software, San Diego California, USA) and the plasma dilution at which 50% neutralization was recorded (IC50) was determined by transforming the data to a log10 scale with fitted sigmoidal dose-response curves. Neutralization breadth of a plasma sample is defined as the percent (0% - 100%) of the 20 isolates neutralized by that sample [14]. All plasma samples were screened for non-HIV-specific neutralization using the murine leukemia virus (MLV) pseudotyped into the HIV backbone.

In certain cases, competition neutralization experiments were performed in the presence of D368R gp120, as previously described in detail [49]. Briefly, serially diluted HIV+ plasmas were pre-incubated with D368R (25 µg/ml) for 1 hour at 37°C and then the mixture was incubated with virus for another hour at 37°C, and subsequently with cells as described above. The fold decrease in log10 IC50 neutralization titers of each plasma tested against each virus in the presence of D368R gp120 was determined.

## Results

### Plasma Neutralizing Activities of Subject AC053

**Figure 1:**
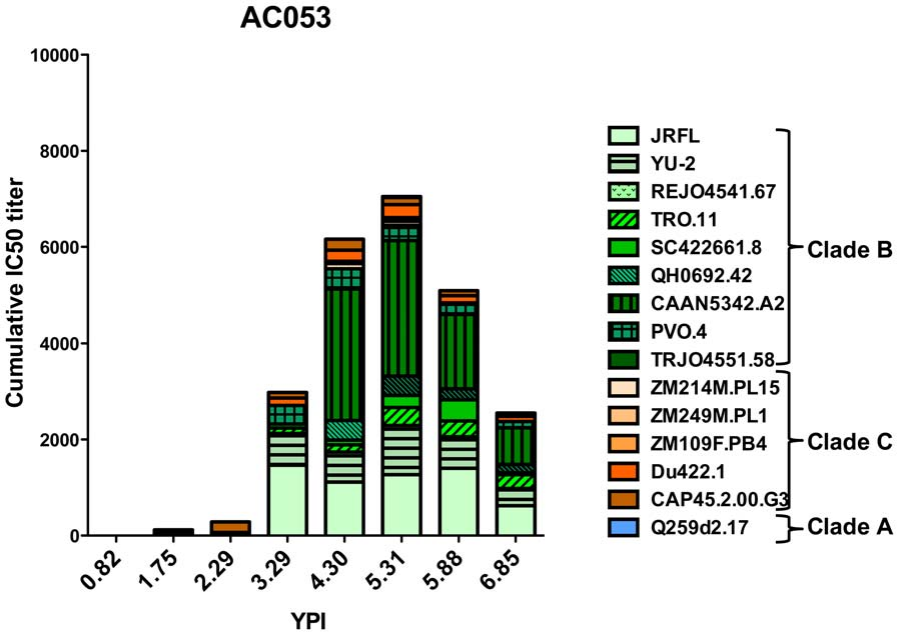
Cumulative IC50 titers of AC053 longitudinal plasma samples as previously reported [14]. The IC50 neutralizing plasma antibody titers against 19 heterologous Clade A (blue), B (green), and C (orange) isolates were determined at distinct time points during infection. The sum of these titers (cumulative IC50 titer) is shown. The neutralizing antibody response gradually increased in breadth and potency, and at the highest recorded breadth (5.31 ypi), AC053 neutralized 16 of these isolates (80% breadth). The most potent neutralizing activities were against Clade B isolates.

To determine how the epitope specificities of the broadly neutralizing antibody response evolved over time we focused our analysis on a single subject in the MGH cohort - AC053. Yearly plasma samples were available for AC053 starting at 0.82 years to 6.85 years post infection, after which the subject initiated antiretroviral therapy. Previously, we characterized the development of the broadly neutralizing antibody response in this subject and demonstrated that at 3.29 years this subject’s plasma could neutralize 45% of the 20 cross-clade isolates tested, and at 5.31 years –80% [14]. The cumulative IC50 titers at all time-points (the sum of the reciprocal dilutions to achieve 50% inhibition of infectivity) are shown in Figure 1. Initial epitope-mapping studies demonstrated that the broadly neutralizing antibody response of AC053 at 5.31 years was primarily focused on the CD4-BS as present on monomeric gp120 [14]. However, for some isolates tested (for example TRO.11, CAAN, and Zm214M), neutralizing activity of the plasma was unaffected by the depletion of anti-gp120 or anti-MPER antibodies. This led us to hypothesize that at least one additional antibody specificity was present in that subject’s plasma; a specificity that targeted an epitope on the virion-associated Env, outside the CD4-BS. The availability of frequent and long-term available samples made this subject a convenient case to study the evolution of dual epitope specificities of the cross-reactive antibody response to HIV.

### Kifunensine-treated Viruses are No Longer Neutralized by AC053 Plasma

**Figure 2:**
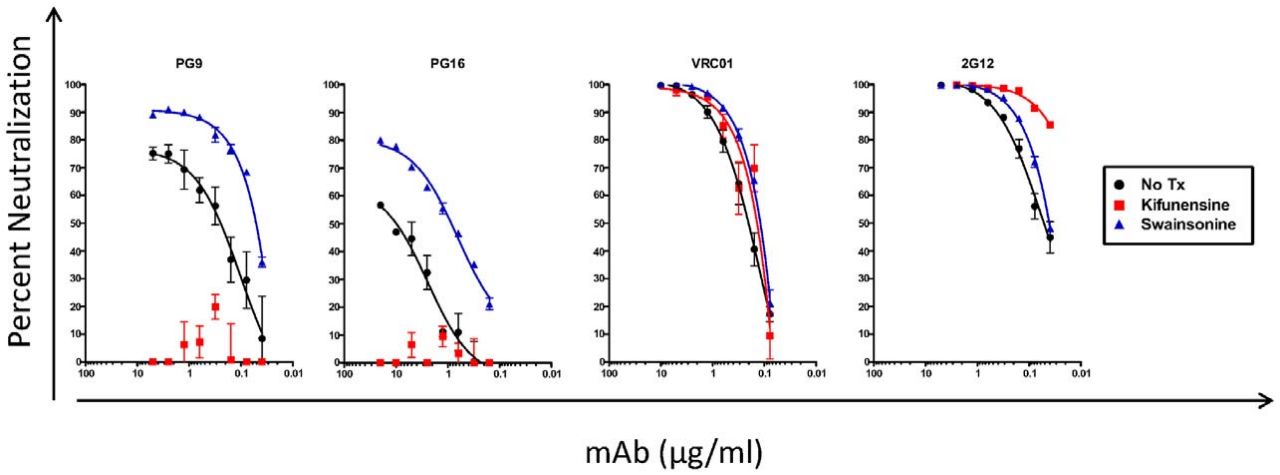
Neutralization of kifunensine- and swainsonine-treated virions by monoclonal antibodies. Neutralization curves were plotted for MAbs PG9, PG16, VRC01 and 2G12 with untreated (black circles), kifunensine-treated (red squares), and swainsonine-treated (blue triangles) **SC422661 pseudovirus**.

MAbs PG9, PG16 and CH01-04 represent a class of cross-neutralizing specificities that primarily target the virion-associated Env over corresponding soluble recombinant Env forms, and map outside the CD4-BS [36], [50]. These types of antibodies recognize a complex epitope within the V2 loop that is formed both by amino acids and glycan molecules [35]. MAbs PGT125-128 represent a second class of cross-neutralizing specificities that also recognize two conserved glycans on gp120 but target a complex epitope that includes the amino acid backbone of the V3 loop [37], [38]. A known distinctive feature of the PG9/PG16 epitope-like specificities (as compared to the PGT-like epitope specificities) is the loss of neutralizing activity against kifunensine-treated viruses [36], [51]. Kifunensine is a mannose analogue that inhibits type-I alpha-glycosidases, and HIV virions produced by kifunensine-treated cells are resistant to neutralization by PG9 and PG16 [51]. However, this treatment does not affect substantially the neutralization of other broadly neutralizing mAbs, such as VRC01 or 2G12 (Figure 2) and others [29]. In addition, the neutralizing activity of the PGT125-128 MAbs (which recognize a conserved epitope in the V3 loop and associated carbohydrates) is not affected by such treatment [38]. As a negative control we included treatment with swainsonine, an enzyme that inhibits mannosidase-II, which others have reported that it does not abrogate neutralization by PG9 and PG16 [51]. Interestingly, swainsonine-treated HIV was more susceptible to PG9, PG16 and 2G12 than the untreated virus (Figure 2).

**Figure 3:**
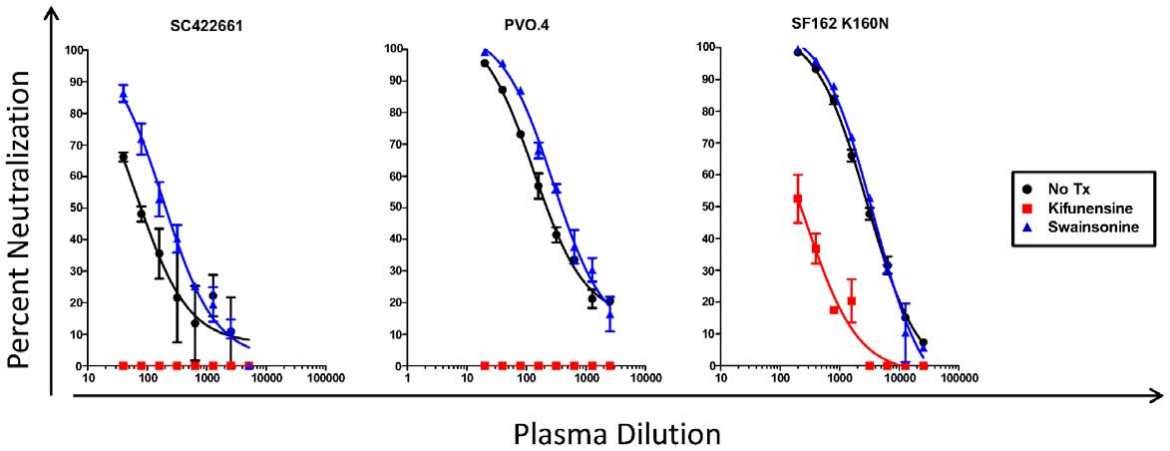
Neutralization of kifunensine- or swainsonine-treated viruses by AC053 plasma collected at 5.31 yrs post infection. The percent neutralization of viruses produced in 293 T cells either in the presence or absence of 20 µM kifunensine or swainsonine glycosidase inhibitors (as described in the Materials and Methods) was determine at a given plasma dilution. Black circles – no treatment, red squares – kifunensine treatment, blue triangles – swainsonine treatment.

We initially examined whether PG9/PG16-like neutralizing activities were present in AC053 plasma, by comparing the neutralizing activity of this plasma using viruses produced in the absence or presence of kifunensine. PG9/16-like antibodies do not recognize the SF162 gp120 because it lacks glycans at position 160, which are necessary for PG9/16-Env binding [36], [52]. This would therefore explain why the anti- TRO.11, CAAN, or Zm214M neutralizing activity of AC053 plasma could not be eliminated by SF162gp120-based antibody adsorptions [14]. The introduction of an asparagine at that position (SF162K160N) renders the virus highly susceptible to PG9/16 [53]. Therefore, we tested AC053 plasma at 5.31 yrs PI against SC422661, PVO.4 and SF162 K160N viruses grown in the presence or absence of kifunensine or swainsonine (Figure 3). Neutralizing activity against all kifunensine-treated viruses was either completely absent (SC422661 and PVO.4) or markedly decreased (SF162K160N) compared to untreated or swainsonine-treated viruses. This result suggested that, potentially, the AC053 plasma contained PG9/16-like antibodies.

**Figure 4:**
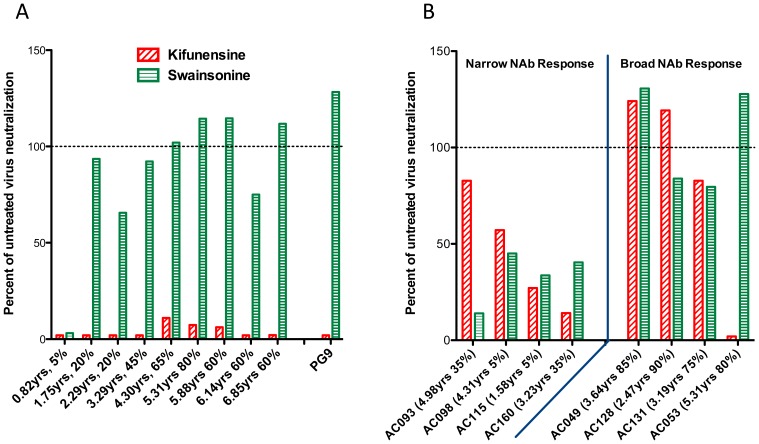
Glycan-dependent neutralizing specificity of plasma samples. Percent change in the plasma IC50 titers of kifunensine-treated (red) and swainsonine-treated (green) SF162 K160N was calculated relative to the untreated virus. Years post infection and percent isolates neutralized are indicated for each sample. (A) Yearly longitudinal AC053 plasma samples from 1.75 years to 6.85 years demonstrated sensitivity to kifunensine treatment only, while the 0.82 samples could not neutralize either treated virus. PG9 is included as a positive control. (B) Plasma samples from the MGH cohort, characterized in detail in a previous report [14], were tested for neutralization of treated and untreated viruses. Four plasmas with narrow breadth (≤35% breadth) and four plasmas with cross-reactive NAb responses (≥75% breadth) were selected. AC053 was the only plasma that demonstrated the PG9-like resistance of kifunensine-, but not swainsonine-treated pseudoviruses.

The above analysis of AC053 was performed with plasma collected at 5.31 years post infection, at a time when the plasma broadly neutralizing activities in this subject were well established. To determine how early this specificity emerged in the plasma of AC053, and whether it coincided with the emergence of the overall broadly neutralizing activity in this subject, we performed similar studies with plasmas collected longitudinally. The earliest samples, however, do not display broadly neutralizing activities and do not neutralize SC422661 or PVO.4 [14], and therefore we could not use those viruses for this experiment. All samples, however, do neutralize the SF162K160N virus. The neutralizing activities of longitudinal plasmas from AC053 were evaluated against SF162K160N grown in the presence or absence of kifunensine (Figure 4A). The earliest plasma (collected at 0.82 yrs after infection) could not neutralize either the kifunensine- or swainsonine-treated viruses. In contrast, plasma collected at 1.75 yrs post-infection could only neutralize the untreated virus and the swainsonine-treated virus, but not the kifunensine-treated virus. These results suggest that, potentially, PG9/16-like neutralizing activities began emerging in this subject within the first two years of infection, sometime between 0.82 and 1.75 yrs post-infection (at the same time as the overall cross-neutralizing activity of AC053 plasma began to be detectable [14]). This relatively early development of PG9/16-like antibodies during HIV infection was recently reported in other HIV+ subjects [16], [26]. AC053 plasmas collected after that point of infection also neutralized the WT virus and the swainsonine-treated virus, but not the kifunensine-treated virus. Therefore, the kifunsensine-dependent neutralizing specificity in AC053 was maintained for the duration of the observation (over 6 years of infection).

In light of these results, we wanted to investigate how common the kifunensine-dependent neutralizing specificity was in the MGH cohort. The effect of kifunensine treatment was tested on plasmas from 3 other subjects from that cohort that developed broadly neutralizing antibody responses and on plasmas from 4 subjects from that cohort that did not develop such responses (Figure 4B). In contrast to the results obtained with the AC053 plasma, kifunensine treatment did not have the same effect on the neutralizing activities of these 7 plasmas. Our findings suggested, therefore, that the sensitivity to kifunensine but not swainsonine was a unique feature in the AC053 broadly neutralizing antibody response in this cohort. Of note, another study of a distinct cohort of chronically HIV-1-infected subjects with broad NAb responses reported a similar frequency of PG9/16-like neutralizing antibody specificities [26].

### Depletions of PG9-like Neutralizing Activities from AC053 Plasma

**Figure 5:**
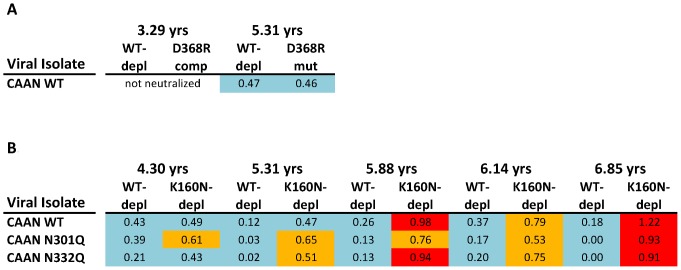
Contribution of different anti-gp120 specificities for the cross-reactive neutralizing activity of AC053 plasma. ( A) Log10 decrease in IC50 titers by elimination of anti-WT SF162 gp120 or competition of anti-D368R SF162 gp120 antibodies, as reported in [14], which demonstrates the CAAN is not susceptible to the anti-CD4BS cross-reactive NAb responses of AC053. (B) Log10 decrease in IC50 titers by elimination of anti-WT SF162 gp120 or anti-K160N SF162 antibodies, indicative of a glycan-specific cross-neutralizing response. Light blue: no effect or less than 0.5 Log10 decrease; Orange: decrease between 0.5 and 0.9 Log10; Red: over 0.9 Log10 decrease.

We next tested the hypothesis that the glycan-specific, PG9-like cross-neutralizing activity in AC053 plasma would be ‘depleted’ if appropriate reagents are used. To this end, we performed the following set of experiments. Plasmas collected at four time-points after the development of broadly neutralizing antibody responses were depleted from their anti-gp120 antibodies with either WTSF162gp120 (which does not deplete PG9-like antibodies) or SF162K160Ngp120 (which depletes PG9-like antibodies) (Figure S1). Both Envelope proteins deplete anti-CD4-BS antibodies (data not shown). The neutralizing activities of the undepleted and depleted plasmas were evaluated and compared using CAAN-derived viruses as targets (Figure 5). CAAN is one of the viruses that, although susceptible to the neutralizing activity of AC053 plasma, is not susceptible to the anti-CD4-BS antibodies present in AC053 (Figure 5A and [14]). Aside from the WT CAAN, two variants of CAAN were also included in these experiments. One (CAAN N301Q) lacks the N-linked glycosylation site at the N-terminus of the V3 loop at position 301, and the other (CAAN 332) lacks the N-linked glycosylation site at the C-terminus of the V3 loop. These two glycans are part of the epitopes of the PGT-like antibodies [37], [38]. The high-mannose glycan at position 332 is also part of the 2G12 epitope – another glycan-dependent MAb [54].

The neutralizing activities of the AC053 plasmas were not affected by the WTSF162gp120 depletion treatment (Figure 5B). However, the SF162K160Ngp120 depletion resulted in incremental loss of the neutralizing activity of AC053 plasmas. The IC50 neutralizing titers of AC053 plasma increased (i.e., less potent neutralization) by more than half a Log 10, initially against CAAN N301Q (4.30 ypi), then against CAAN N301Q and CAAN N332Q (5.31 ypi), and finally against all three viruses tested (CAAN N301Q, CAAN N332Q and CAAN) (5.88 ypi).

### Relative Emergence of Broadly Neutralizing Antibody Responses to the CD4-BS and to the Env Carbohydrates in AC053

**Figure 6:**
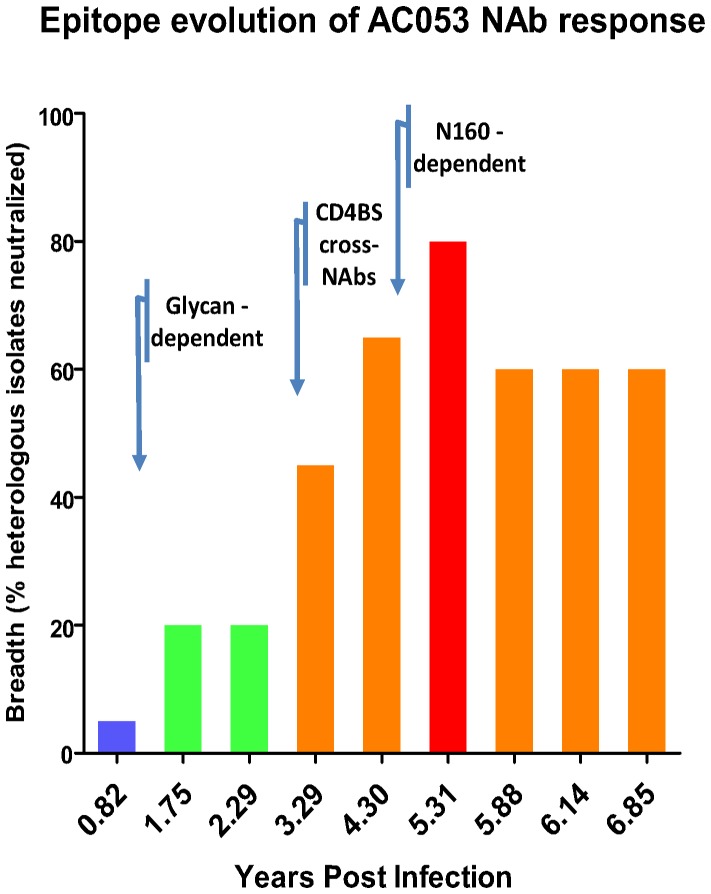
Timeline of the epitope evolution of cross-reactive NAb responses in AC053. The breadth of neutralizing antibody responses (i.e., the percent of heterologous isolates neutralized by plasma samples out of the total isolates tested [14]), was plotted for all available time-points for subject AC053. The arrows on the timeline correspond to approximate years post infection when particular neutralizing antibody specificities became evident. Breadth is color-coded as follows: blue 0–19%, green 20–39%, orange 40–74%, red 75–100%.

Our results indicate that the glycan-dependent neutralizing activity in the plasma of AC053 emerged sometime between 0.82 and 1.75 ypi (Figure 5). At the time this activity became evident (at 1.75 ypi), the overall breadth of neutralizing activity in the blood was very narrow with ∼20% of heterologous clade B and C isolates tested being susceptible to neutralization by AC053 plasma [14]. At approximately 3 ypi, anti-CD4-BS antibodies with cross-neutralizing potential became detectable (Figure 6 and [14]). As mentioned above, and in our previous study [14], these antibodies were capable of neutralizing a wide range of isolates, but not every isolate tested against (for example, TRO or CAAN). The neutralizing activity that was dependent on the presence of glycans at position 160 became evident much later, sometime after 4.30 ypi. We believe that PG9-like antibodies, rather than PGT-like antibodies or 2G12-like antibodies, emerged at that time in AC053, because: (a) this neutralizing activity is affected by the kifunensine treatment of the target virus, (b) it is dependent on the presence of carbohydrates at position 160, and (c) is independent on the presence of carbohydrates at positions 301 or 332.

## Discussion

In the past few years several groups reported that approximately 15% of those infected with HIV-1 develop broadly neutralizing antibody responses [12]–[18]. MAbs with potent and broad anti-HIV neutralizing activities have been isolated from such subjects [31], [32], [36], [38]–[40], [50]. We and others have shown that broadly neutralizing antibody responses become detectable in HIV+ plasmas on average at 2 to 3 years post infection, but very rarely before that [14], [16], [19]. Our initial epitope-mapping analysis indicated that, similar to what has been observed in studies of chronic HIV infection [25], [26], a relatively small number of Env regions are targeted by the earliest cross-neutralizing neutralizing antibody responses [14].

The characterization of such MAbs has provided new information on the structure of broadly neutralizing anti-HIV-1 antibodies, the location and structures of their epitopes, and has expanded our knowledge of the mechanisms by which such antibodies prevent HIV-infection [31], [33]–[35], [55]–[58]. Whether the overall broadly neutralizing activity of an individual’s plasma is due to a single or multiple antibody epitope specificities remains a topic of intensive investigation. Recent studies, conducted with samples collected from a very small number of HIV-1-infected subjects, indicate that one or two distinct epitope specificities can recapitulate the majority of the broadly neutralizing activity of the corresponding plasma [42], [43], [59]. In one report, the anti-CD4-BS broadly neutralizing activity was complemented by a PG9/16-like neutralizing activity [42], while in the other report the anti-CD4-BS broadly neutralizing activity was complemented by an activity targeting a not well-defined epitope that is preferentially exposed once Env engages CD4 [43]. This information is very relevant not only to future HIV vaccine-design efforts, but also to our understanding of the way the human immune system (specifically its B cell arm) responds to HIV infection. The above observations were, however, made in the context of chronic HIV infection and it remains unknown whether dual specificity broadly neutralizing antibody responses emerge at the same time of infection, or sequentially, in a given subject.

To address the topic of the relative timing of emergence of broadly neutralizing antibody responses that target two (or more) distinct Env regions within a given subject, we focused our attention on patient AC053. The clade B-infected AC053 was part of a cohort of HIV-1-infected subjects that were monitored longitudinally from the time of their HIV-1 infection [14]. We previously reported that the broadly neutralizing antibody activity of this subject’s plasma was due to at least two specificities: one that targets the CD4-BS on gp120 and was effective against many, but not all, isolates the plasma was tested against, and another specify that was not targeting the CD4-BS or the gp41 Env subunit [14]. The second specificity was effective against viruses that were naturally resistant to this subject’s anti-CD4-BS NAbs. The availability of nine plasma samples from AC053 collected between 0.82 to 6.85 years post infection, and the knowledge of when plasma broadly neutralizing antibody responses became detectable in this individual, made it a perfect case for studying the evolution of dual epitope specificities of broadly neutralizing antibody responses during HIV-1 infection.

Sometime between 0.82 and 1.75 years of infection a glycan-dependent neutralizing activity became apparent in the blood of AC053. The timing of the emergence of this activity coincides with the initial appearance of cross-neutralizing antibodies in this subject. At 0.82 ypi the plasma only neutralized SF162 (out of 20 isolates tested), while at 1.75 ypi the plasma neutralized four out of 20 isolated tested [14]. Two of these four isolates were clade C and two were clade B. At that point, however, the potency of neutralization was weak and the breadth of neutralization was narrow. In addition, several isolates that are susceptible to PG9 were resistant to neutralization by this plasma. Overall, these observations suggested to us that, at its earliest development, the glycan-dependent neutralizing activity in AC053 plasma was not due to PG9-like antibodies. Of course, one could also argue that PG9-like antibodies began emerging at that point of infection, but that their VH and VL antibody domains had not yet incurred somatic mutations that are required for the broad neutralizing ability of PG9. In the absence of longitudinally isolated MAbs from AC053 it is not possible to address this point directly. Broader cross-neutralizing antibody responses capable of neutralizing at least 50% of isolates tested (from clades A, B and C) became first apparent at approximately 3 ypi and were due to anti-CD4-BS neutralizing antibodies (Figure 6 and [14]). As we extensively discussed previously, these anti-CD4-BS cross-neutralizing activities were not effective against all isolates that were susceptible to neutralization by the AC053 plasma [14]. For example, they were not effective against the CAAN or TRO.11 viruses. Even the anti-CD4-BS neutralizing activities of plasmas isolated later in infection, which were broader and more potent, were ineffective against these and other viruses. At 3 ypi, cross-neutralizing specificities that are dependent on the presence of a glycan at position 160 were not evident in AC053. This second cross-neutralizing specificity became apparent sometime around 4.30 ypi. Because of its dependency on the 160 glycan but not on glycans positioned in regions of Env targeted by the PGT-like antibodies or 2G12-like antibodies, we believe that this second cross-neutralizing specificity is due to PG9-like antibodies. We do not believe it is due to PG16-like antibodies, because the neutralizing activity of PG16 cannot be blocked by SF162K160N gp120, while that of PG9 and of the AC053 plasma antibodies are efficiently blocked by that recombinant protein.

We used two independent methods to demonstrate the presence of a PG9-like glycan-dependent epitope specificity of the broadly neutralizing antibody response in AC053. The use of glycosidase inhibitors, such as kifunensine, to enrich high mannose glycans is a well-established method and has been previously used to identify glycan-dependent epitopes targeted by anti-HIV antibody responses [26], [29], [51]. Of note, the nature of the glycosylation pattern on HIV Env can be influenced by the host cell and culture conditions used [60], [61]. The majority of studies on antibody responses to HIV have used pseudoviruses produced in cell lines, such as the 293T used in this study. However, it is possible that these viruses have different N-linked glycosylation pattern than the ones replicating in infected hosts. Consequently, viruses grown in T cells treated with kifunensine could potentially have different susceptibility to neutralization than the ones produced by cell lines. Despite these caveats, the studies with glycosidase inhibitors described here provided reproducible and interpretable results that were in agreement with the other experimental strategy used – depletion with a SF162 K160N glycoprotein.

Our results are in agreement, and extend, those recently reported by Bonsignori et al [42] that two distinct broadly neutralizing antibody specificities, one against the CD4-BS and the other against the V1V2 loop (PG9-like), can be simultaneously present in an HIV+ subject with broad serum neutralizing activities. The timing of relative emergence of these two distinct specificities was unknown in that study. Here we independently show similar dual specificities in a different subject, but we also report that the anti-CD4-BS specificity emerged first, while the PG9-like specificity emerged approximately 1–2 years later. It remains unknown whether these two types of specificities can be generated simultaneously in an HIV-infected subject and whether they can be elicited simultaneously by vaccination. We do not currently have viral sequence information on AC053 to define how the virus evolved in that subject before and after the development of the anti-CD4-BS neutralizing antibodies. The V2 loop is positioned in a way that limits the accessibility of the CD4-BS to neutralizing antibodies [62]–[64]. Thus, it is possible that in its effort to escape their action, the virus altered the amino acid composition and/or glycosylation pattern of the V2 loop, and such escape viral env clones were the ones that stimulated B cells to produce PG9-like antibodies. We believe that the earliest development of glycan-dependent neutralizing specificities aided in the development of PG9-like antibodies later in infection.

In summary, our studies support efforts to elicit broadly neutralizing anti-HIV antibodies of multiple epitope specificities by vaccination. Most likely this will be achieved by prime-boost immunization protocols, during which sequential immunizations with distinct Env proteins will stimulate the development of broadly NAbs of distinct epitope specificities.

## Supporting Information

Figure S1 **Depletion of PG9 neutralizing activity with SF162 K160N gp120.** PG9 in naïve human sera was depleted with 4 consecutive incubations with SF162K160N gp120-coupled beads, as discussed in the Materials and Methods section. Neutralization by undepleted (solid symbols and lines) and depleted (clear symbols and dashed lines) PG9 was tested against SF162 K160N (orange circles) and REJO (blue squares) viruses, demonstrating the substantially diminished neutralization activity upon depletion.
(TIF)
