# Supplementary figures and images for: Evolution of Cross-Neutralizing Antibody Specificities to the CD4-BS and the Carbohydrate Cloak of the HIV Env in an HIV-1-Infected Subject
Source: PLoS One. 2012 Nov 13;7(11):e49610. doi: 10.1371/journal.pone.0049610 (PMC3496710; doi:10.1371/journal.pone.0049610)

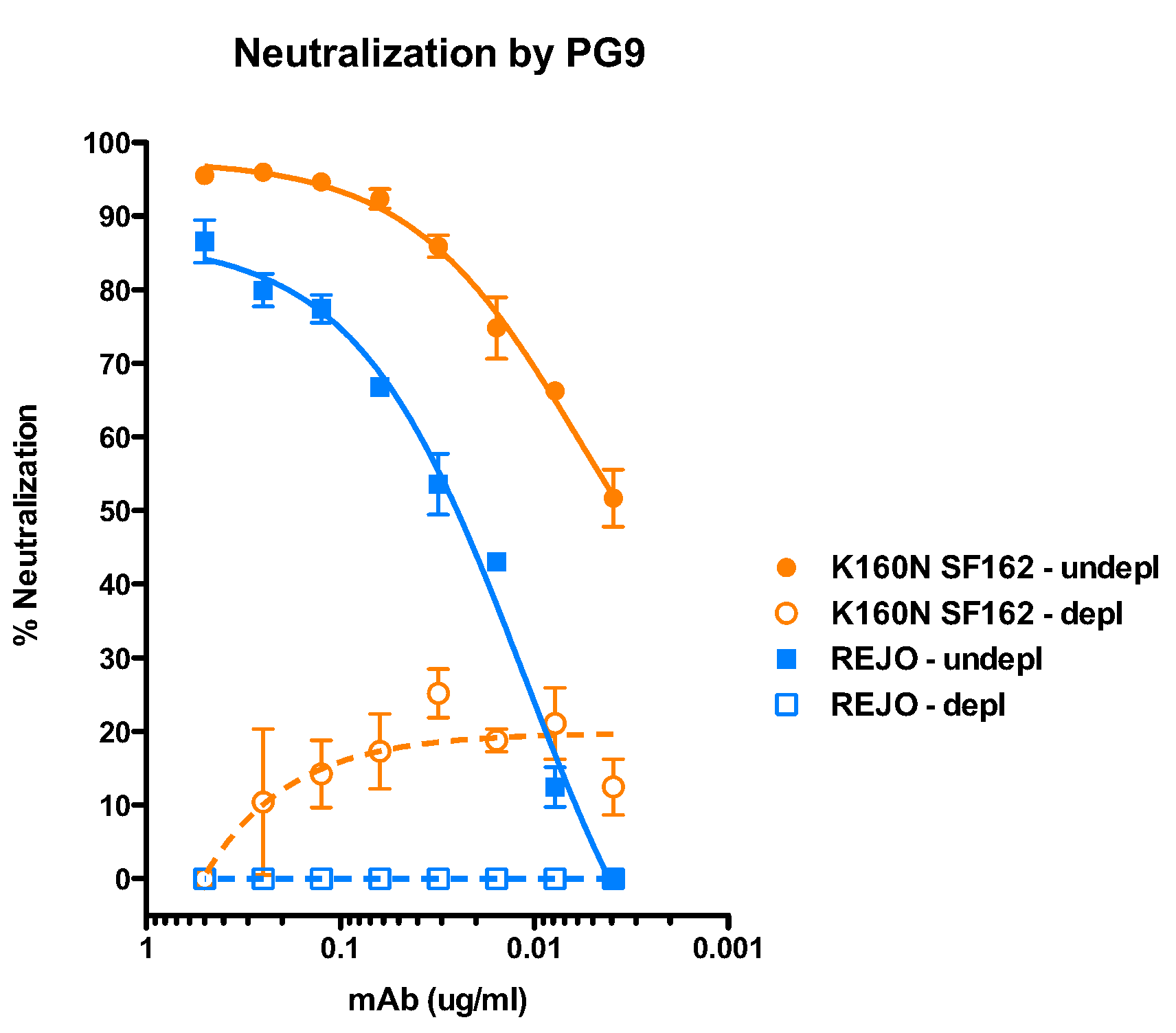

Supplement: Figure S1 — Depletion of PG9 neutralizing activity with SF162 K160N gp120. PG9 in naïve human sera was depleted with 4 consecutive incubations with SF162K160N gp120-coupled beads, as discussed in the Materials and Methods section. Neutralization by undepleted (solid symbols and lines) and depleted (clear symbols and dashed lines) PG9 was tested against SF162 K160N (orange circles) and REJO (blue squares) viruses, demonstrating the substantially diminished neutralization activity upon depletion. (TIF) [file pone.0049610.s001.tif]
